# No evidence for transmission of psychosis, bipolar or depressive disorder via hematopoietic stem cell transplantation: A Swedish registry study

**DOI:** 10.1111/pcn.13448

**Published:** 2022-08-09

**Authors:** Lucas D. Ekström, Simon Pahnke, Gunnar Larfors, Hans Hägglund, Mats Lekander, Gustaf Edgren, Simon Cervenka

**Affiliations:** ^1^ Department of Medicine Solna, Clinical Epidemiology Unit, Karolinska Institutet Stockholm Sweden; ^2^ Department of Medical Sciences Uppsala University Uppsala Sweden; ^3^ Stress Research Institute, Department of Psychology Stockholm University Stockholm Sweden; ^4^ Department of Clinical Neuroscience Karolinska Institutet Stockholm Sweden; ^5^ Department of Medical Sciences, Psychiatry Uppsala University Uppsala Sweden; ^6^ Centre for Psychiatry Research, Department of Clinical Neuroscience Karolinska Institutet and Stockholm Health Care Services, Region Stockholm Stockholm Sweden

Two case reports have triggered the hypothesis that risk for psychiatric disorders can be transmitted through hematopoietic stem cell transplantation (HSCT) by transfer of a dysregulated immunological phenotype; one case of development of severe psychosis in a patient with no prior psychiatric history following HSCT for chronic lymphatic leukemia from his sibling diagnosed with schizophrenia,^1^ and one case of remission of psychosis in a patient with treatment‐resistant schizophrenia following HSCT for acute myeloid leukemia.^2^ We evaluated the possibility that donors' risk of psychosis, bipolar and depressive disorder can be transmitted to the recipient in a nationwide cohort of donors and recipients who underwent HSCT.

We used an approach previously applied to study transfusion‐transmitted disease using large‐scale register data.^3^, ^4^ All related donors and recipients who underwent HSCT in Sweden between 1977 and 2014 were identified. Linkage with a range of nationwide registers provided clinical details and follow‐up until December 31st, 2015 for all HSCT recipients and their donors together with non‐donor first degree relatives of the recipients. Exposure was defined as receiving stem cells from a donor who was diagnosed with psychosis, bipolar disorder or depression either before HSCT or during follow‐up (F20‐39 in ICD‐10; 295–299, 311 in ICD‐9; 295–299 in ICD‐8). Outcome in the recipient was defined as the same diagnoses during follow‐up, which was extended from time of HSCT until death, emigration or end of follow‐up. Recipients diagnosed with any of the three psychiatric disorders prior to HSCT were excluded. The hazard ratio of developing each psychiatric disorder in recipients in relation to whether the donor was affected by the same disorder was estimated using three separate Cox proportional‐hazards regression models. All models included a number of possible confounding variables, ascertained for recipients at the time of HSCT: sex (nominal), age (continuous), disposable income (continuous), educational level (ordinal) and county of residence (nominal). We also included binary terms for family history of the same psychiatric diagnoses included as outcome, as derived from non‐donor first degree relatives of the recipients, using a similar approach as in previous studies on transfusion ‐ transmission.^3^, ^4^ 95% confidence limits for the ensuing hazard ratios were constructed using heteroscedasticity‐consistent standard errors. The study was approved by the regional ethical review boards in Stockholm and Uppsala (1998/259 and 2016/497, respectively).

A total of 1363 HSCT donor‐recipient pairs were included (1262 siblings, 28 paternal donors, 20 maternal donors and 53 donors from other relatives), together with 5466 non‐donor first degree relatives (2856 parents and 2610 non‐donor siblings). 658 of the recipients deceased during the study period (mean survival time 2.79 years), the remaining 705 were followed up for a mean of 13.14 years. 16 donors were diagnosed with psychosis, nine with bipolar disorder and 81 with depression, before or after HSCT. During the follow‐ period of 11,074 person years in total (median 4.4 years), 10 of the recipients were diagnosed with psychosis, seven with bipolar disorder and 68 with depression (Table 1). None of the 16 and 9 HSCT recipients whose donors were diagnosed with psychosis or bipolar disorder, respectively, were diagnosed with either disorder (Table 1). Among the 81 HSCT recipients who received a stem cell transplant from a donor with depressive disorder, nine were diagnosed with depression. This corresponded to an unadjusted hazard ratio of 2.5 (95% CI, 1.3–5.1). However, when adjusting for the full range of covariates, including history of any of the studied psychiatric disorders in non‐recipient first degree relatives, the risk estimate attenuated to an adjusted hazard ratio of 1.8 (95% CI, 0.6–5.3). Findings persisted across all exposures and outcomes irrespective of whether the diseased donor received their diagnosis before or after the HSCT (data not shown).

**Table 1:** Association between occurrence of psychiatric disorder in donor and risk of the same disorder in transplant recipients, stratified by disease outcome

Disease outcome	Number of patients	Events/person‐years	Unadjusted hazard ratio (95% CI)	Adjusted hazard ratio (95% CI)
Psychosis				
Donor diseased	16	0/140	0.0^†^	0.0^†^
Donor not diseased	**1347**	**10/10 934**	**1.0 (ref)**	**1.0 (ref)**
Bipolar disorder				
Donor diseased	9	0/81	0.0^†^	0.0^†^
Donor not diseased	**1 354**	**7/10 993**	**1.0 (ref)**	**1.0 (ref)**
Depression				
Donor diseased	81	9/609	2.5 (1.3–5.1)	1.8 (0.6–5.3)
Donor not diseased	**1 282**	**59/10 465**	**1.0 (ref)**	**1.0 (ref)**

^†^ Where there were no events during follow‐up, hazard ratios (unadjusted and adjusted) and 95% confidence intervals are represented as zero above. Alternatively, by using the longstanding “rule of threes” principle of unknown origin^5^, the corresponding estimates would be 0.2 (0.0–4.0) and 0.4 (0.0–4.0) for psychosis and bipolar disorder respectively.

The potential transmission of psychiatric disease through HSCT could reflect a possible causative role of the immune system in the modulation of risk for psychiatric illness.^1^, ^2^ To our knowledge, the present work represents the first attempt to systematically investigate this possibility. A transfer of risk for psychosis, bipolar disorder or depression could not be confirmed. The main limitation is restricted power due to low incidence of psychiatric disorders in our sample, as well as the short survival time following HSCT, meaning that the sensitivity for true effects is low. Moreover, the resolution of the data did not allow for an analysis on improvement or recovery in patients with psychiatric illness following HSCT. We welcome an international effort to pool HSCT register data to replicate our analysis using a larger study population.

## Disclosure statement

All authors declare no conflicts of interest.

## Author contributions

Conception and design of the study: S.C., L.E., S.P., G.E., M.L.; acquisition and analysis of data: L.E., S.P., G.L., H.H.; drafting the manuscript: L.E., S.C. All authors interpreted the results, critically revised the manuscript and approved of the final version for publication.
